# Response of a Bell–Bloom Magnetometer to a Magnetic Field of Arbitrary Direction

**DOI:** 10.3390/s18051401

**Published:** 2018-05-02

**Authors:** Zhichao Ding, Jie Yuan, Xingwu Long

**Affiliations:** College of Advanced Interdisciplinary Studies, National University of Defense Technology, Changsha 410073, China; dingzhichao13@nudt.edu.cn (Z.D.); jieyuan@nudt.edu.cn (J.Y.)

**Keywords:** Bell–Bloom magnetometer, frequency response, linewidth

## Abstract

The Bell–Bloom magnetometer in response to a magnetic field of arbitrary direction is observed theoretically and experimentally. A theoretical model is built from a macroscopic view to simulate the magnetometer frequency response to an external magnetic field of arbitrary direction. Based on the simulation results, the magnetometer characteristics, including the signal phase and amplitude at resonance, the linewidth, and the magnetometer sensitivity, are analyzed, and the dependencies of these characteristics on the external magnetic field direction are obtained, which are verified by the experiment.

## 1. Introduction

In order to realize the effective detection of an external magnetic field, many kinds of magnetometers, like the fluxgate [1], Hall probe [2], proton magnetometer [3], soft ferromagnetic dot arrays [4], superconducting quantum interference device (SQUID) [5,6], and atomic magnetometer [7,8], appeared one after another. Of these magnetometers, the SQUID is the most sensitive magnetometer which has been commercially produced, but it needs a cumbersome refrigeration device. Unlike the SQUID, the atomic magnetometer can achieve comparable sensitivity with non-cryogenic operation. Because of this, the atomic magnetometer is widely regarded as the most ideal option in many significant fields, such as medicine [9], tests of fundamental symmetries [10], space exploration [11], and detection of nuclear magnetic resonance signals [12,13,14].

The atomic magnetometer detects an external magnetic field by measuring the coherent precession frequency of atomic spins about the external magnetic field [7,8]. For realizing the coherent precession of atomic spins, two main kinds of atomic magnetometers, the radio-optical magnetometer and the Bell–Bloom magnetometer, respectively apply a radio-frequency magnetic field and modulated light to excite the atomic magnetic dipole transition and create the transverse spin component, with respect to the external magnetic field direction [15,16,17]. Each of these two magnetometers has its own characteristics. Compared with the radio-optical magnetometer, the Bell–Bloom magnetometer does not need to produce the radio-frequency magnetic field, so it can be applied in some areas which expect to avoid the interference of an additional field, such as tests of fundamental symmetries [18], and can be miniaturized [19].

Since the Bell–Bloom magnetometer was proposed by Bell and Bloom [17], it has been attracting wide attention. Many aspects of the Bell–Bloom magnetometer were studied and discussed by researchers, ranging from the basic principle [17], the characteristics of the signal amplitude and phase [20,21], to the influence of the pump light on the magnetometer performance [20,21,22]. Researchers have realized the high-bandwidth Bell–Bloom magnetometer [23], as well as the miniaturized Bell–Bloom magnetometer [19], and investigated their characteristics. In addition, the idea of the synchronous optical pumping of the Bell–Bloom magnetometer was also studied and adopted, to improve the performance of the magnetometer based on nonlinear magneto-optical rotation [24]. In these studies of the Bell–Bloom magnetometer, an external magnetic field is applied under magnetic shield, and the typical experimental condition is that the external magnetic field is parallel or perpendicular to the pump light direction [19,20,21,22,23,25], though the dependency of the signal amplitude on the angle between the propagation direction of the pump light and the external magnetic field was also discussed [21,22]. However, when the Bell–Bloom magnetometer is put into practice eventually, the situation that the external magnetic field is in an arbitrary direction is a general case.

When the Bell–Bloom magnetometer is put into practice eventually, in order to choose the proper detection method, the dependency of the signal phase on the external magnetic field direction should be known, and for obtaining a better understanding of the magnetometer performances, like the dead zones and the sensitivity, the dependencies of the signal amplitude and the linewidth on the external magnetic field direction need to be investigated. However, these aspects are rarely studied. Therefore, it is necessary to make a deeper research on the characteristics of the Bell–Bloom magnetometer in response to an external magnetic field of arbitrary direction. In this paper, we choose ^133^Cs atoms as the sensory atoms for near room-temperature operation, theoretically and experimentally observing the response of a Bell–Bloom magnetometer to a magnetic field of arbitrary direction.

## 2. Theory and Simulation

**Figure 1:**
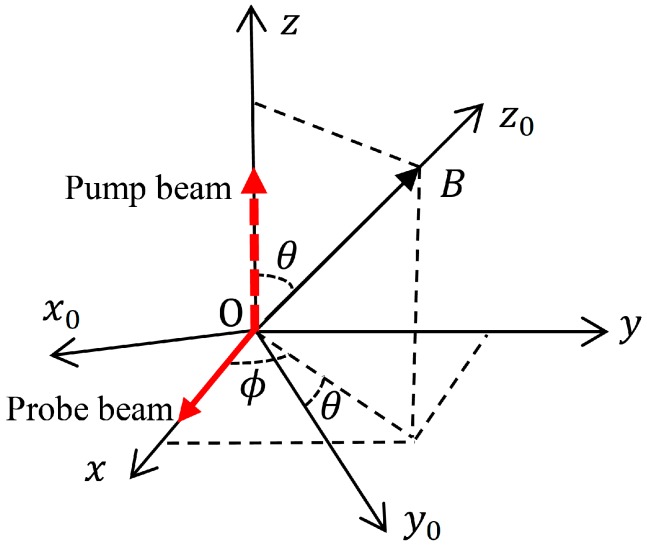
Reference frames of the studied Bell–Bloom magnetometer.

As shown in Figure 1, the *z*_0_-axis is along an arbitrary direction in the laboratory reference frame xyz, and its polar and azimuth angles are θ and ϕ, respectively. Considering a vapor cell which contains ^133^Cs atoms and buffer gas in an external static magnetic field $B{\hat{z}}_{0}$, where ${\hat{z}}_{0}$ is the unit vector along the *z*_0_-axis, the ^133^Cs atomic spins, which can be described by a spin polarization vector P, will precess about the *z*_0_-axis from a macroscopic view. When the density of ^133^Cs atoms is low, so that the atomic system is not in the spin-exchange relaxation free regime [8], considering the spin relaxation due to some relaxation mechanisms [26], and neglecting the spin polarization of ^133^Cs atoms in the lower ground-state hyperfine level, since it is much smaller than that of ^133^Cs atoms in the upper ground-state hyperfine level in the general case and under our experimental condition [8,27,28], the evolution of P, which is represented as $\left(P_{x0},\;P_{y0},\;P_{z0}\right)$ in the laboratory reference frame x_0_y_0_z_0_, satisfies the following Bloch equation [27,28]:

(1) $$\frac{dP}{dt}=\gamma B{\hat{z}}_{0}\times P-\frac{P_{x_{0}}{\hat{x}}_{0}+P_{y_{0}}{\hat{y}}_{0}}{T_{2}}+\frac{P_{0}-P_{z_{0}}}{T_{1}}{\hat{z}}_{0}.$$

Here, γ is the gyromagnetic ratio of the ^133^Cs atomic spin, $T_{2}$ and $T_{1}$ are respectively the transverse and longitudinal spin relaxation times of ^133^Cs atoms, and $P_{0}$ is the steady spin polarization for this system. ${\hat{x}}_{0}$ and ${\hat{y}}_{0}$ are respectively the unit vectors along the x_0_ and y_0_ axes, and ${\hat{x}}_{0}$ is in the x–y plane as shown in Figure 1.

For the Bell–Bloom magnetometer, a circularly polarized pump beam with amplitude modulation is applied to polarize the atomic ensemble [29]. Assume that the pump beam propagates along the *z*-axis as shown in Figure 1, and its intensity is $I_{0}\left[\cos(\omega t)+1\right]/2$, where $I_{0}$ is the maximum instantaneous pump light intensity, and ω is the modulation frequency. The pump light attempts to improve the spin polarization of ^133^Cs atoms along the y_0_ and z_0_ axes [28,29]. Meanwhile, the absorption of pump light by the ^133^Cs atoms randomizes the direction of atomic spins, relaxing the transverse and longitudinal spin components [26,28]. Adding the influences of optical pumping, Equation (1) becomes

(2) $$\begin{array}{l} \frac{dP}{dt}=\gamma B{\hat{z}}_{0}\times P-\frac{P_{x_{0}}{\hat{x}}_{0}+P_{y_{0}}{\hat{y}}_{0}}{T_{2}}+\frac{P_{0}-P_{z_{0}}}{T_{1}}{\hat{z}}_{0}+R_{op}\frac{\cos(\omega t)+1}{2}(-\sin\theta {\hat{y}}_{0}+\cos\theta {\hat{z}}_{0})\\ \;\;-R_{rel\_op}\frac{\cos(\omega t)+1}{2}(P_{x_{0}}{\hat{x}}_{0}+P_{y_{0}}{\hat{y}}_{0}+P_{z_{0}}{\hat{z}}_{0}) \end{array}.$$

Here, $R_{op}$ is the maximum instantaneous optical pumping rate, and $R_{rel\_op}$ is the maximum instantaneous spin relaxation rate due to the absorption of pump light. Compared with the spin polarization achieved by optical pumping, $P_{0}$ is much smaller, and can be ignored. Therefore, Equation (2) can be rewritten as

(3) $$\left\{ \begin{array}{l} \frac{dP_{x_{0}}}{dt}=-\left[\frac{1}{T_{2}}+R_{rel\_op}\frac{\cos(\omega t)+1}{2}\right]P_{x_{0}}-\omega_{0}P_{y_{0}}\\ \frac{dP_{y_{0}}}{dt}=\omega_{0}P_{x_{0}}-\left[\frac{1}{T_{2}}+R_{rel\_op}\frac{\cos(\omega t)+1}{2}\right]P_{y_{0}}-R_{op}\frac{\cos(\omega t)+1}{2}\sin\theta \\ \frac{dP_{z_{0}}}{dt}=-\left[\frac{1}{T_{1}}+R_{rel\_op}\frac{\cos(\omega t)+1}{2}\right]P_{z_{0}}+R_{op}\frac{\cos(\omega t)+1}{2}\cos\theta \end{array}\right.,$$

where $\omega_{0}=\gamma B$ is the magnetic resonance frequency.

When the conventional pump-probe scheme is used to extract the coherent precession frequency for the Bell–Bloom magnetometer, a linearly polarized probe beam propagating along the *x*-axis is applied to detect the x-component $P_{x}$ of the spin polarization [23,25]. According to Figure 1, one can easily obtain

(4) $$P_{x}=\sin\phi P_{x_{0}}+\cos\phi \cos\theta P_{y_{0}}+\cos\phi \sin\theta P_{z_{0}}$$

**Figure 2:**
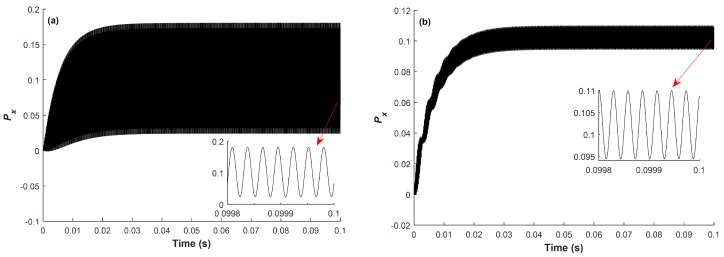
Two simulation results of the x-component of the spin polarization at (**a**) $\omega =\omega_{0}$ and (**b**) $\omega =0.99\omega_{0}$. The simulation conditions are as below: θ=π/3, ϕ=π/4, $T_{1}=10\;\mathrm{ms}$, $T_{2}=6\;\mathrm{ms}$, $R_{op}=R_{rel\_op}=100{\;s}^{-1}$, and $\omega_{0}=2\pi \times 35\;\mathrm{kHz}$.

Based on Equations (3) and (4), $P_{x}$ can be numerically simulated using Mathematica software. For observing the characteristics of $P_{x}$, when the modulation frequency is equal to or deviates from the magnetic resonance frequency, $P_{x}$ at $\omega =\omega_{0}$ and $\omega =0.99\omega_{0}$ are simulated, and the results are shown in Figure 2a,b, respectively. We can find from Figure 2 that, compared with the situation when $\omega =0.99\omega_{0}$, the modulated pump light at $\omega =\omega_{0}$ can create a much larger transverse spin component with respect to $B{\hat{z}}_{0}$, due to the magnetic resonance. After a period of several times of $T_{2}$, this system will reach a steady state, and $P_{x}$ will oscillate at the modulation frequency with fixed amplitude, and can be represented as

(5) $$P_{xr}\sin(\omega t+\psi )+P_{x0}=P_{xc}\cos(\omega t)+P_{xs}\sin(\omega t)+P_{x0}.$$

Here, $P_{xr}=\sqrt{{P_{xc}}^{2}+{P_{xs}}^{2}}$, $\psi =\arctan(P_{xc}/P_{xs})$, $P_{xc}$, $P_{xs}$ and $P_{x0}$ are the steady values of the amplitude, phase, in-phase component, quadrature component, and dc component of $P_{x}$, respectively.

**Figure 3:**
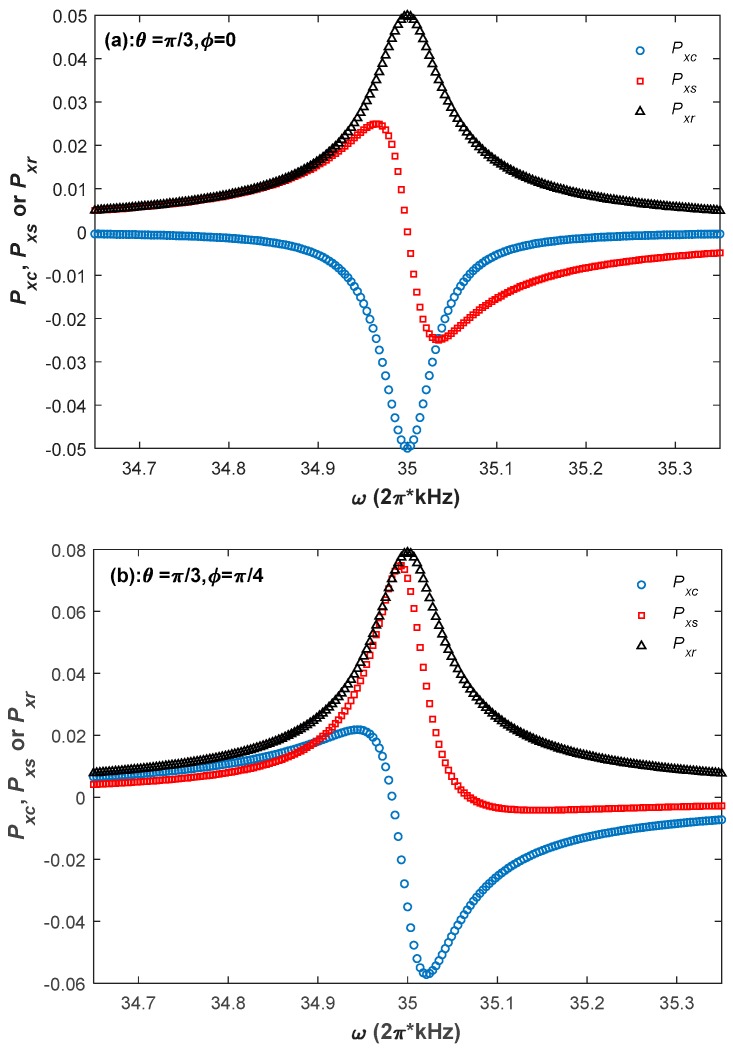
Two simulation results of the frequency response spectrums at (**a**) θ=π/3, ϕ=0 and (**b**) θ=π/3, ϕ=π/4. The simulation conditions are as below: $T_{1}=10\;\mathrm{ms}$, $T_{2}=6\;\mathrm{ms}$, $R_{op}=R_{rel\_op}=100{\;s}^{-1}$, and $\omega_{0}=2\pi \times 35\;\mathrm{kHz}$.

For estimating the performance of the Bell–Bloom magnetometer, the magnetometer frequency response to the modulated pump light is simulated. Figure 3 shows two simulation results of the frequency response spectrums when the external magnetic field is in two different directions. The blue circles, red squares, and black triangles in Figure 3 represent the simulation values of $P_{xc}$, $P_{xs}$ and $P_{xr}$, respectively. As shown in Figure 3a, similar to the conventional situation that the external magnetic field is perpendicular to the pump light direction, the magnetometer frequency response also consists of a dispersive component and an absorption component [8,25]. However, when the direction of the external magnetic field changes, $P_{xs}$ is no longer a standard dispersion signal, and $P_{xc}$ is no longer a standard absorption signal, as shown in Figure 3b, demonstrating that ψ is different when the external magnetic field is in these two directions.

**Figure 4:**
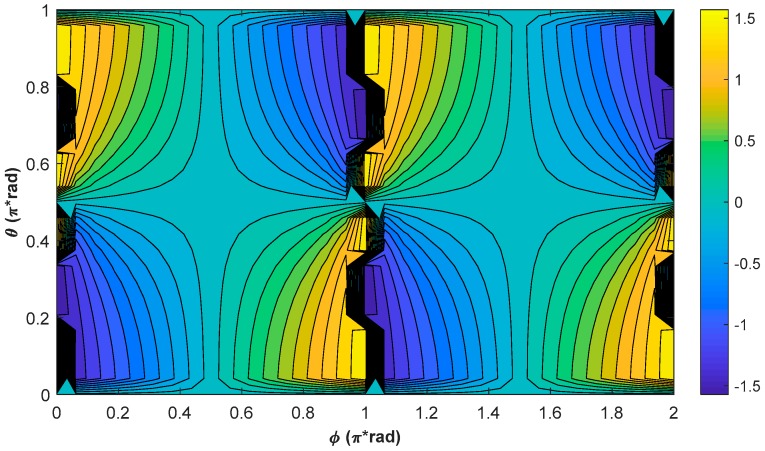
Contour plot of the simulation results of ψ at resonance. The simulation conditions are as below: $T_{1}=10\;\mathrm{ms}$, $T_{2}=6\;\mathrm{ms}$, $R_{op}=R_{rel\_op}=100{\;s}^{-1}$, and $\omega_{0}=2\pi \times 35\;\mathrm{kHz}$.

Figure 4 shows the contour plot of the simulation results of ψ at resonance when the external magnetic field is in different directions. As shown in Figure 4, ψ varies with ϕ for a fixed θ except for θ=π/2, and varies with θ for a fixed ϕ, except for ϕ=π/2 or 3π/2, further showing that ψ varies with the direction of the external magnetic field. In addition, using the same method, one can also simulate the steady values of the phases of $P_{y}$ and $P_{z}$ at resonance, and can find that they vary with the direction of the external magnetic field as well.

For the atomic magnetometer, the synchronous phase detection is a conventional method to extract the magnetic resonance frequency, and further derive the strength of an external magnetic field. Since this detection method regards the zero-crossing frequency of the dispersion signal as $\omega_{0}$ [8], if the demodulation phase does not match the signal phase, the phase error will influence the measuring accuracy of the external magnetic field, as shown in Figure 3. Therefore, if the synchronous phase detection is adopted by the Bell–Bloom magnetometer, there may be a great measuring error, since the signal phase varies with the external magnetic field direction. So, the synchronous phase detection is impractical for the Bell–Bloom magnetometer.

Though the phase mismatch affects the in-phase and quadrature components of $P_{x}$, it has no influence on the amplitude of $P_{x}$. As shown in Figure 3, the central frequencies of $P_{xr}$ are both equal to $\omega_{0}$ even though the external magnetic fields are neither perpendicular to the pump light direction, and the numerical simulation shows that the results are the same when the external magnetic field is in other directions. Therefore, when the Bell–Bloom magnetometer is put into practice for obtaining the strength of an external magnetic field, a practicable method is to extract the central frequency of $P_{xr}$ as the magnetic resonance frequency.

Based on the frequency response spectrum, the response sensitivity of the Bell–Bloom magnetometer to a magnetic field of arbitrary direction can be estimated, since the magnetometer sensitivity is negatively related to the linewidth of the frequency response spectrum, and positively related to the signal amplitude at resonance [8]. As the absorption of pump light by the ^133^Cs atoms relaxes the longitudinal spin component, as well as the transverse spin component, the external magnetic field direction has no influence on the linewidth, in theory. We extract the linewidth by computing the full width at half maximum of the absorption signal when the demodulation phase is set to match the signal phase. The simulation results show that the linewidths are essentially identical within fluctuations of 0.2% for different external magnetic field directions, satisfying the theoretical prediction.

As the atomic spins precess about the external magnetic field from the macroscopic view, to the first order approximation, $P_{z0}$ is a constant value at the steady state, while $P_{x0}$ and $P_{y0}$ oscillate at the modulation frequency with the same amplitude. So, according to Equations (4) and (5), the signal amplitude $P_{xr}$ is proportional to $\sqrt{\left(\sin^{2}\phi +\cos^{2}\theta \cos^{2}\phi \right)}$. In addition, as the transverse spin components, $P_{x0}$ and $P_{y0}$, with respect to the external magnetic field direction, are excited and created by the component of the modulated pump light perpendicular to the external magnetic field direction [8,25], $P_{xr}$ is related to sinθ. Therefore, the dependence of the signal amplitude at resonance on the external magnetic field direction can be approximately represented as

(6) $${P_{xr}|}_{\omega =\omega_{0}}\propto \sin\theta \sqrt{\left(\sin^{2}\phi +\cos^{2}\theta \cos^{2}\phi \right)}.$$

**Figure 5:**
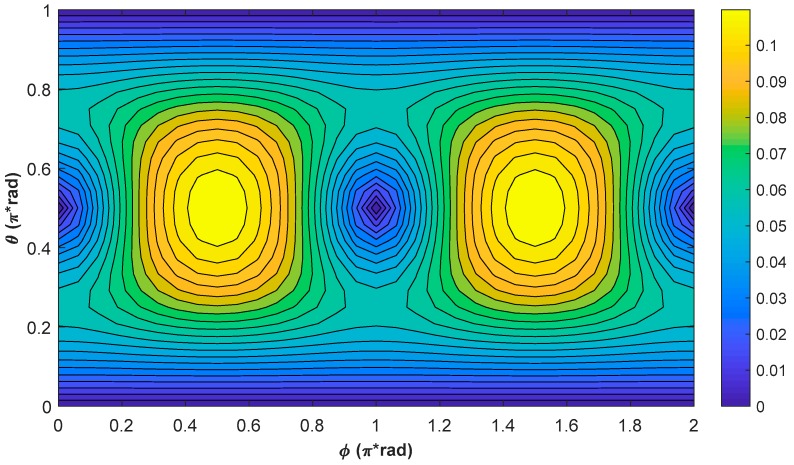
Contour plot of the simulation results of $P_{xr}$ at resonance. The simulation conditions are as below: $T_{1}=10\;\mathrm{ms}$, $T_{2}=6\;\mathrm{ms}$, $R_{op}=R_{rel\_op}=100{\;s}^{-1}$, and $\omega_{0}=2\pi \times 35\;\mathrm{kHz}$.

The relatively precise theoretical values of $P_{xr}$ at resonance can be obtained by extracting the maximum amplitude of the frequency response spectrums, and the contour plot of the simulation results are shown in Figure 5. As shown in Equation (6) and Figure 5, the Bell–Bloom magnetometer does not respond to an external magnetic field, which is along the pump light direction. For a fixed θ, the signal amplitude at resonance reaches the maximum when ϕ=π/2 or 3π/2, and drops to the minimum when ϕ=0 or π. When the external magnetic field deviates from the axis perpendicular to the pump light, the maximum signal amplitude at resonance decreases, and the minimum signal amplitude at resonance increases first when |θ−π/2|<π/4, and then decreases when |θ−π/2|>π/4.

## 3. Experiment and Results

**Figure 6:**
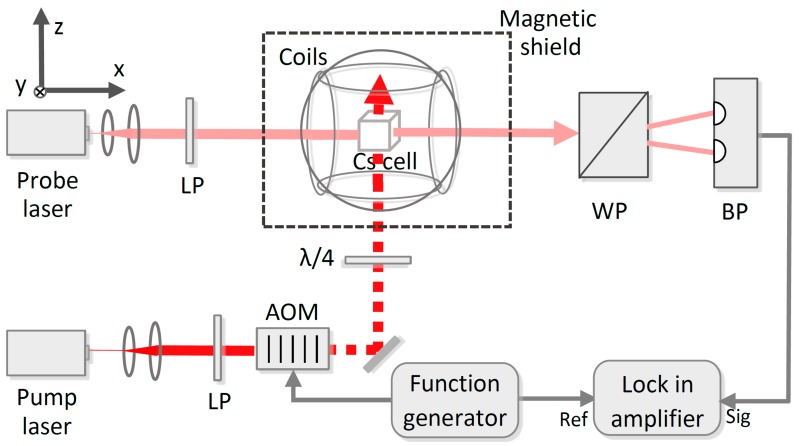
Schematic diagram of the experimental setup. LP: linear polarizer, AOM: acousto-optic modulator, λ/4: quarter-wave plate, WP: Wollaston prism, BP: balanced photodetector.

Figure 6 is the schematic diagram of the experimental setup. ^133^Cs atoms and buffer gas are contained in a cubic cell. The length of the inner side of the cell is 14 mm. In order to prevent the interference of the geomagnetic field, the cell is put in a μ-metal magnetic shield. Two pairs of heating resistors, which are driven by a 10 kHz current, are used to heat the cell. Through feedback control, the temperature of the cell is maintained at 50 °C, and the power of the heating resistors is approximately 1.2 W. The static magnetic field of 10 μT in different directions experienced by the ^133^Cs atoms is generated by three pairs of Helmholtz coils along the x, y, and z axes. The coils are driven by three steady current circuits whose output currents are controlled by a high-precision data acquisition system.

Two distributed feedback diode lasers are employed to generate the pump and probe beams. The intensities of pump light and probe light are, respectively, approximately 400 μW/cm^2^ and 200 μW/cm^2^, and their frequencies are adjusted to the $F=3\to F^{\prime }=4$ component of the ^133^Cs D1 line, and approximately 5 GHz towards upper frequency from the $F=3\to F^{\prime \prime }=4$ component of the ^133^Cs D2 line, respectively. Here, F, $F^{\prime }$, and $F^{\prime \prime }$ indicate the quantum numbers of the total atomic angular momentum when the ^133^Cs atom is in the 6^2^S_1/2_, 6^2^P_1/2_, and 6^2^P_3/2_ states, respectively.

The pump light is first converted to linearly polarized light by a linear polarizer, and modulated by an acousto-optic modulator, which is driven by a function generator. Then, it becomes left circularly polarized light after passing through a λ/4 plate, and polarizes the ^133^Cs atoms along the *z*-axis. The probe light is first converted to the linearly polarized light by a linear polarizer as well. After interacting with the ^133^Cs atoms, the polarization plane of probe light is modulated by $P_{x}$. A Wollaston prism and a balanced photodetector are used to detect the polarization plane of probe light, and the output signal of the balanced photodetector is demodulated by a lock-in amplifier with the reference frequency of the modulation frequency provided by the function generator.

**Figure 7:**
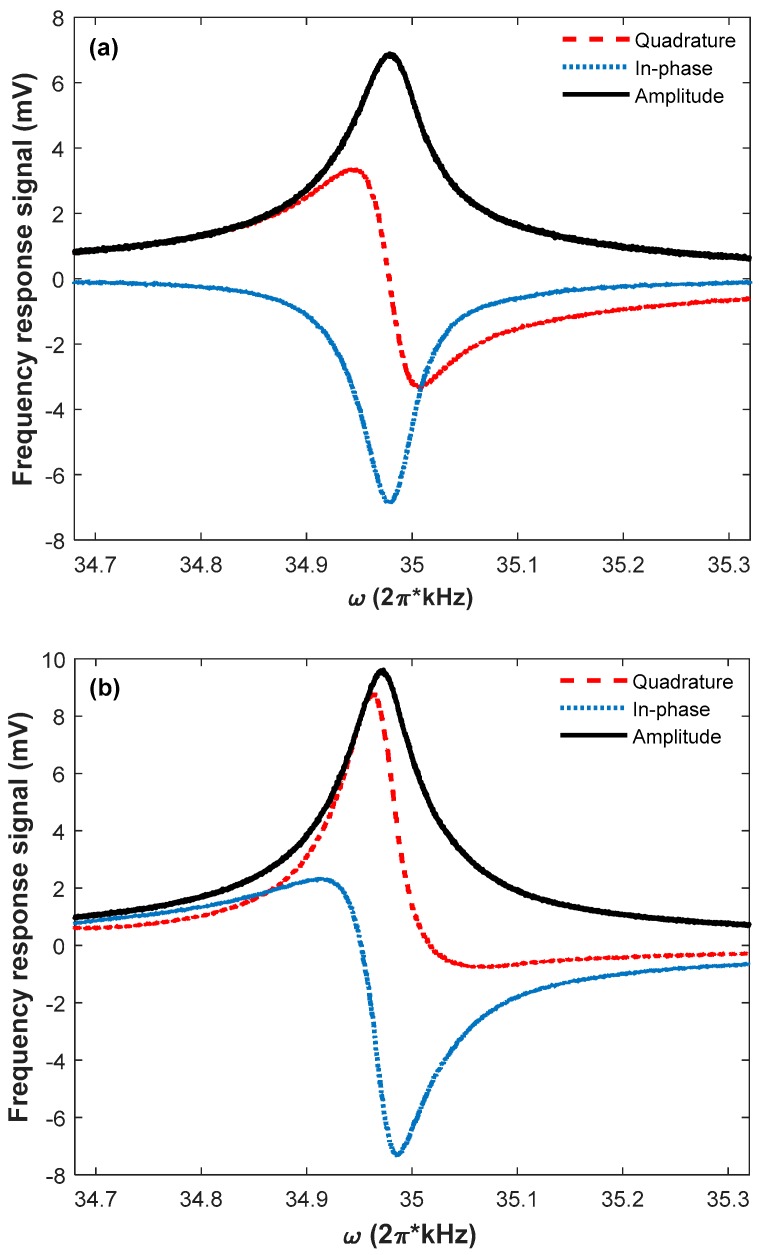
Two experimental results of the frequency response spectrums at (**a**) θ=π/3, ϕ=0 and (**b**) θ=π/3, ϕ=π/4. The red dashed, blue dotted, and black solid lines represent the quadrature signal, the in-phase signal and the signal amplitude, respectively.

In order to observe the characteristics of the Bell–Bloom magnetometer in response to a magnetic field of arbitrary direction experimentally, the magnetometer frequency response spectrums are measured by recording the output in-phase signal, quadrature signal, and signal amplitude of the lock-in amplifier when the modulation frequency is scanned. Figure 7 shows two experimental results of the frequency response spectrums for a fixed demodulation phase when the modulation frequency is scanned at a rate of 10 Hz/s, from 34.7 kHz to 35.3 kHz. The small difference of the central frequencies of the signal amplitudes shown in Figure 7a,b comes from the amplitude error of the applied external magnetic fields. Comparing the results shown in Figure 3 and Figure 7, we find that the experimental results are in a good agreement with the simulation results, verifying that the signal phase varies with the external magnetic field direction, and the synchronous phase detection is impractical for the Bell–Bloom magnetometer. Since the signal amplitude reaches the maximum at resonance, a practicable method is to extract the central frequency of the signal amplitude as the magnetic resonance frequency when the Bell–Bloom magnetometer is put into practice.

**Figure 8:**
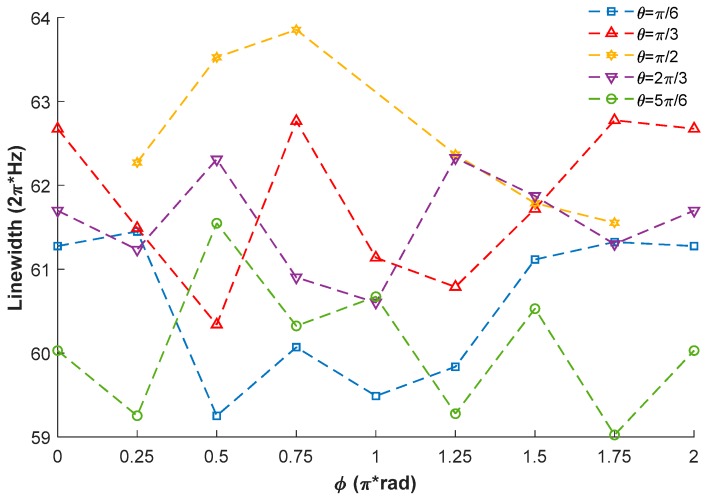
Experimental results of the linewidths of the frequency response spectrums.

When the demodulation phase is adjusted to match the signal phase, the linewidth of the frequency response spectrum can be extracted from the absorption component. The experimental results of the linewidths when the external magnetic field is in different directions are shown in Figure 8. Considering the measuring errors and the fluctuations of the system, the direction of the external magnetic field has little influence on the linewidth, as shown in Figure 8, matching the theoretical prediction.

**Figure 9:**
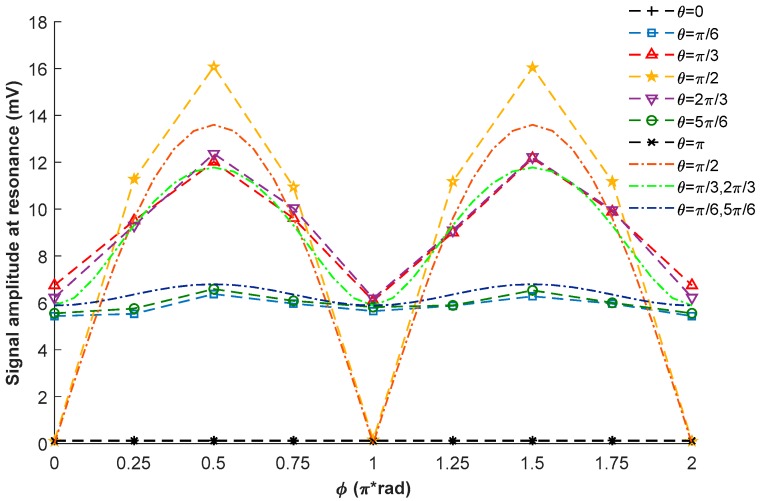
Experimental results (points in the dashed lines) and simulation results (dot-dashed lines) of the signal amplitudes at resonance.

The points in the dashed lines of Figure 9 show the measured signal amplitudes at resonance when the external magnetic field is in different directions, and the dot-dashed lines in Figure 9 are the simulation results of $P_{xr}$ at resonance, which are proportionally enlarged for comparison. As the output signal of the balanced photodetector is not strictly proportional to $P_{x}$ [30], and the simulation result based on the Bloch equation is just an approximation, there are some differences between the experimental and theoretical results. Nevertheless, comparing the results shown in Figure 5 and Figure 9, we can find that the trend in the dependency of $P_{xr}$ at resonance on θ and ϕ can be well predicted by the theoretical simulation.

**Figure 10:**
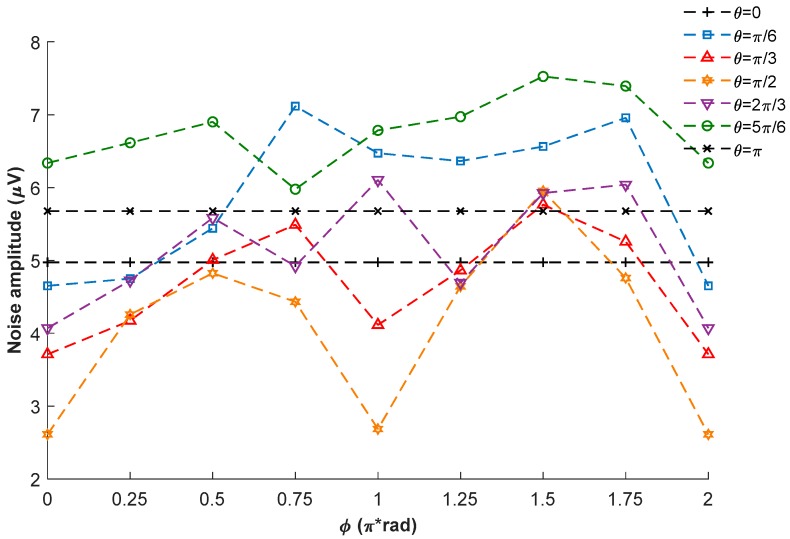
Experimental results of the noise amplitudes at resonance.

Figure 10 shows the measured noise amplitudes when the external magnetic field is in different directions, which are obtained by calculating the root-mean-square values of the signal amplitudes at resonance. Considering the measuring errors and the fluctuations of the system, there is no obvious dependency relation between the noise amplitude and the external magnetic field direction.

Since the external magnetic field direction has little influence on the linewidth and the noise, the influence of the external magnetic field direction on the magnetometer sensitivity is mainly due to its influence on the signal amplitude for the Bell–Bloom magnetometer. Therefore, for the Bell–Bloom magnetometer, the dependency of the magnetometer sensitivity on the external magnetic field direction can also be approximately predicted by $\sin\theta \sqrt{\left(\sin^{2}\phi +\cos^{2}\theta \cos^{2}\phi \right)}$.

## 4. Conclusions

In conclusion, we have theoretically and experimentally observed a Bell–Bloom magnetometer in response to a magnetic field of arbitrary direction. Using the built theoretical simulation model from the macroscopic view, the magnetometer frequency response to a magnetic field of arbitrary direction has been simulated and then verified by the corresponding experiments.

The theoretical and experimental results show that, even though the direction of an external magnetic field is not perpendicular to the pump light direction, the magnetometer frequency response also consists of a dispersive component and an absorptive component when the demodulation phase matches the signal phase. However, the signal phase varies with the external magnetic field direction, making the synchronous phase detection impractical for the Bell–Bloom magnetometer. A practicable detection method is to extract the central frequency of the signal amplitude as the magnetic resonance frequency when the Bell–Bloom magnetometer is put into practice, since the signal amplitude reaches the maximum at resonance.

For the Bell–Bloom magnetometer, the external magnetic field direction has little influence on the linewidth and the noise, and the influence of the external magnetic field direction on the magnetometer sensitivity is mainly due to its influence on the signal amplitude. As a result, the magnetometer sensitivity has an approximately sinusoidal dependency on the azimuth angle of the external magnetic field, and the maximum magnetometer sensitivity decreases when the external magnetic field deviates from the axis perpendicular to the pump light. When the Bell–Bloom magnetometer is put into practice eventually, the above theoretical and experimental conclusions can provide helpful guidance.
